# Supplementary figures and images for: LD-transpeptidation is crucial for fitness and polar growth in Agrobacterium tumefaciens
Source: PLoS Genet. 2024 Oct 21;20(10):e1011449. doi: 10.1371/journal.pgen.1011449 (PMC11527210; doi:10.1371/journal.pgen.1011449)

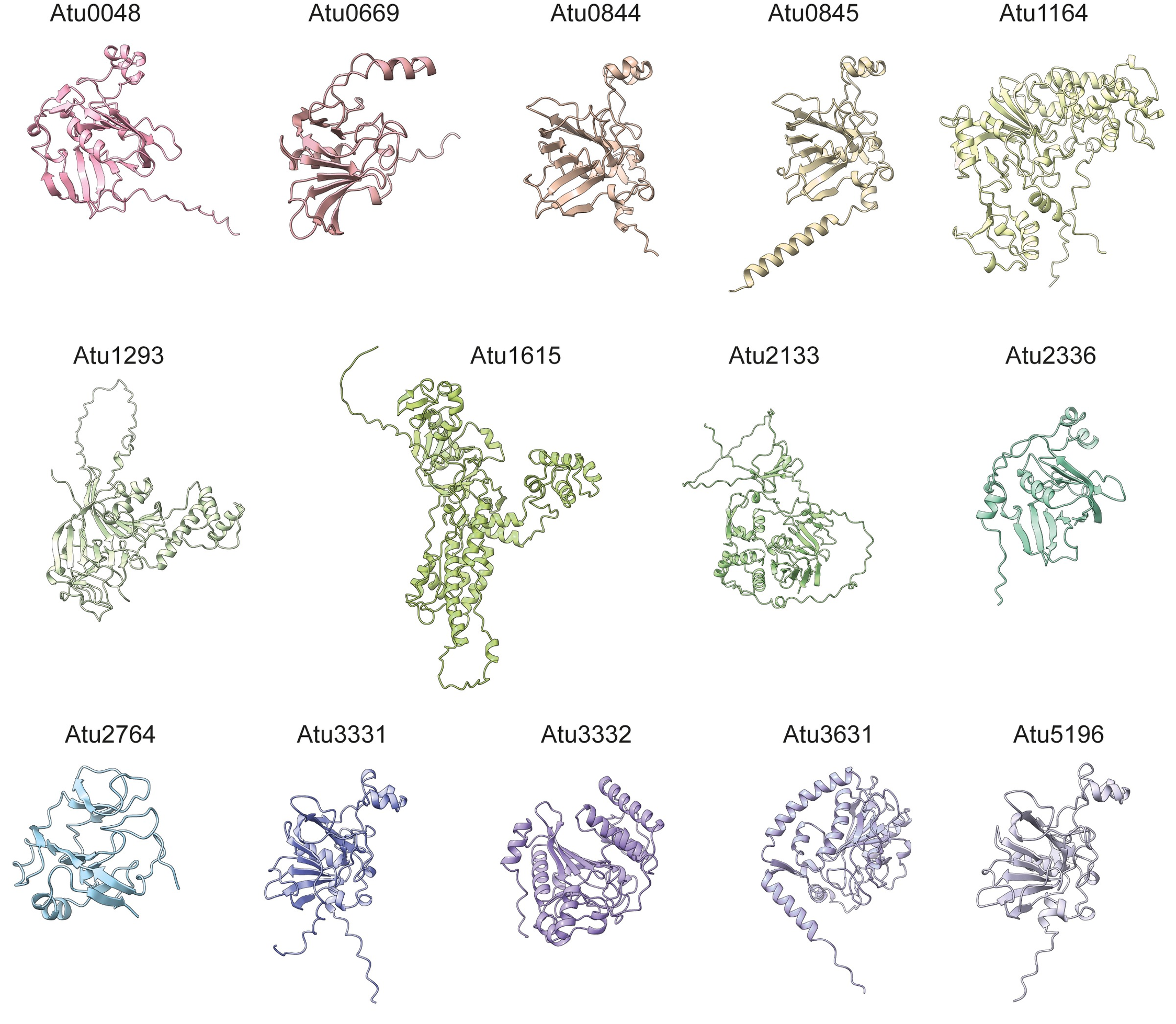

Supplement: S1 Fig — AlphaFold structure models visualized with UCSF ChimeraX. The N- or C-terminal disordered regions with low confidence (predicted local distance difference test, pLDDT, lower than 50) have been hidden. (TIF) [file pgen.1011449.s001.tif]

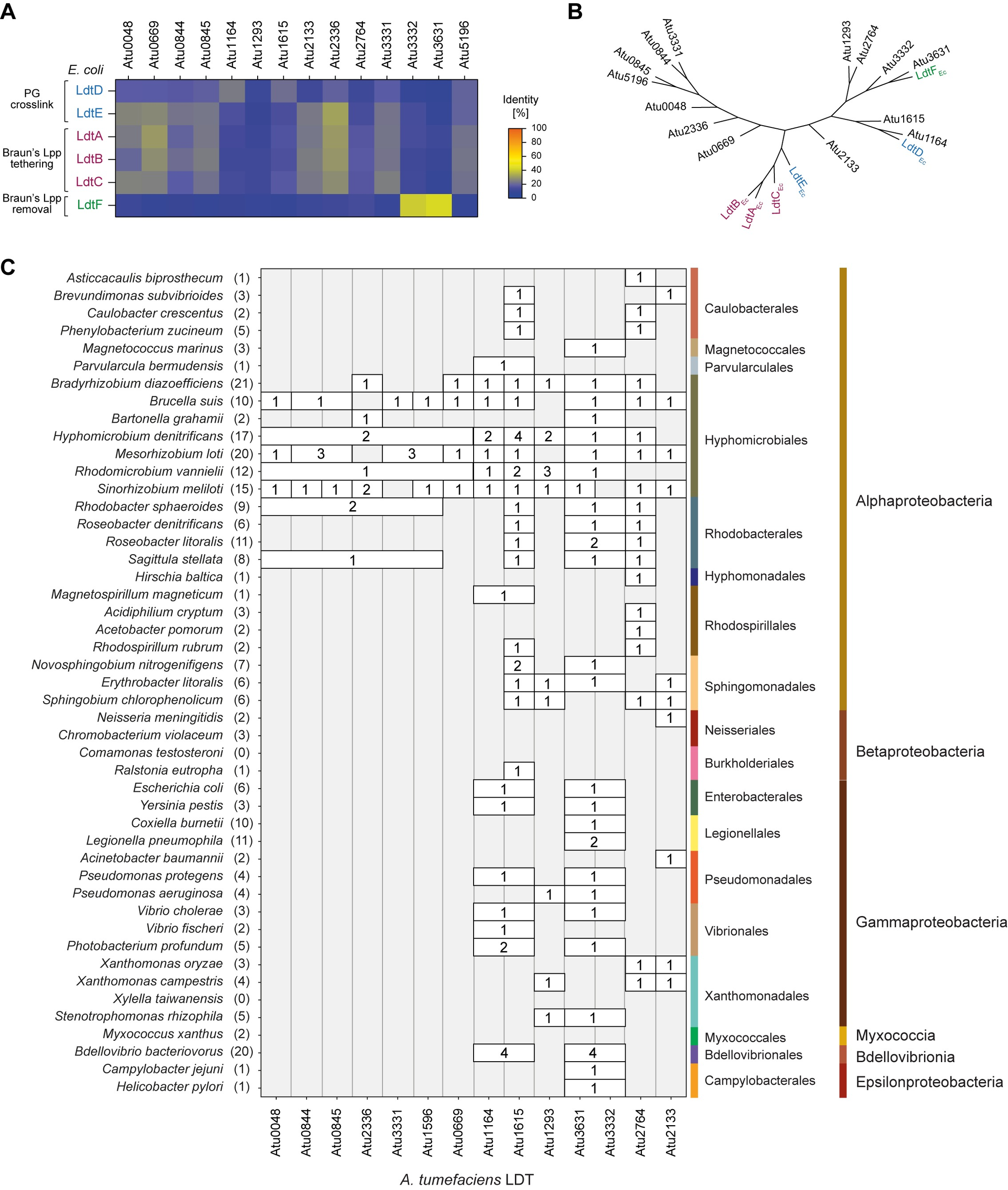

Supplement: S2 Fig — (A) Identity matrix of the 14 LDTs from A. tumefaciens compared to the LDTs in Escherichia coli. (B) Un-rooted phylogenetic tree of the LDTs in A. tumefaciens and E. coli. Protein sequences were aligned using Muscle and the resulting tree was visualized in iTol. (C) Prediction of LDTs number, paralogs and orthologs in other bacteria. Total number of putative LDTs in each species is indicated in brackets. Shared orthologs of A. tumefaciens LDTs are indicated by rectangles spanning related LDTs, the number indicates the copy number of paralogs. (TIF) [file pgen.1011449.s002.tif]

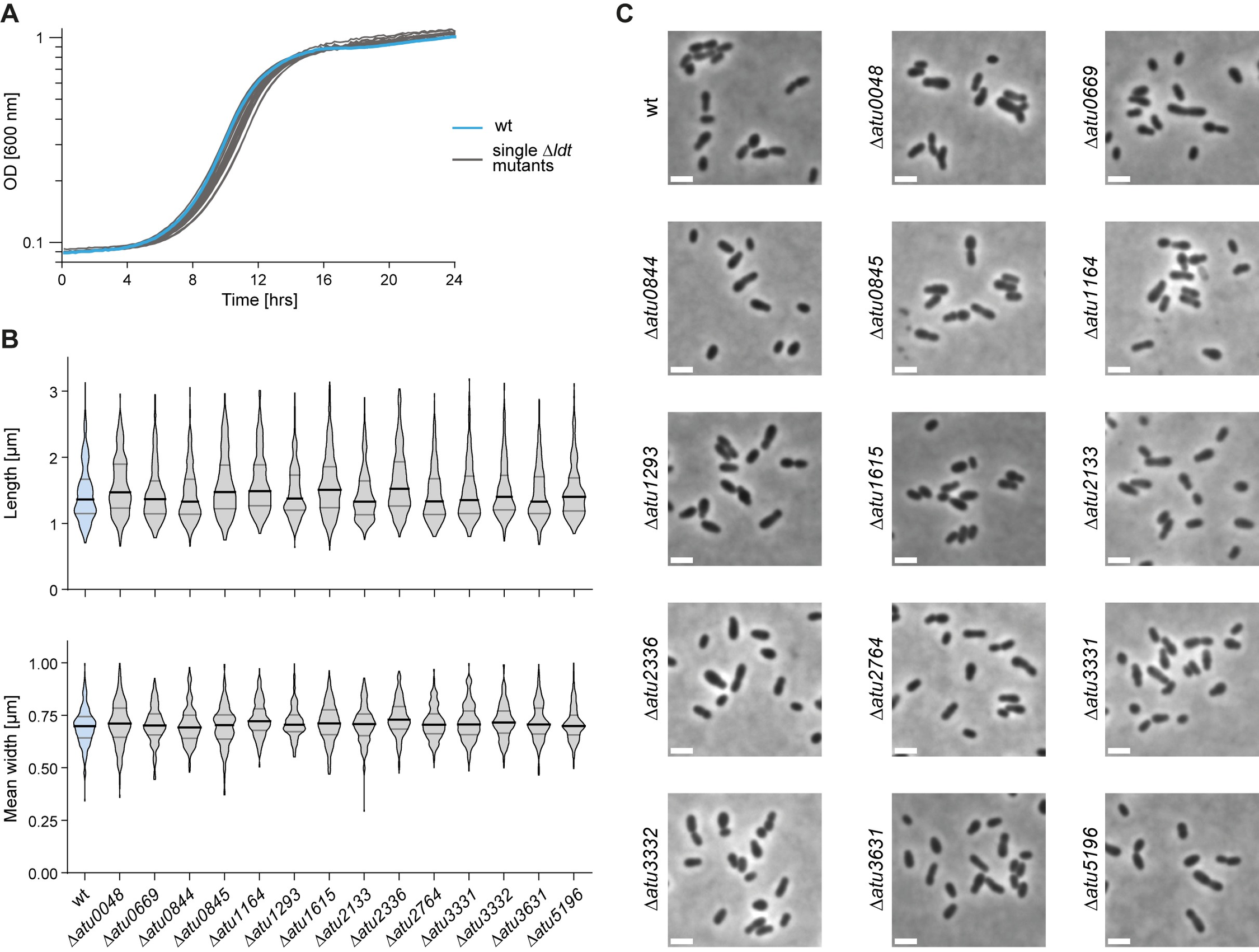

Supplement: S3 Fig — (A) Growth curves of A. tumefaciens wild type (wt) and single Δldt mutants in LB5 (0.5% NaCl) medium. (B) Violin plots of the length and mean width of A. tumefaciens wt and single Δldt mutants grown in LB5. (C) Representative phase contrast images of A. tumefaciens wt and single Δldt mutants grown in LB5. Scale bar: 2 μm. (TIF) [file pgen.1011449.s003.tif]

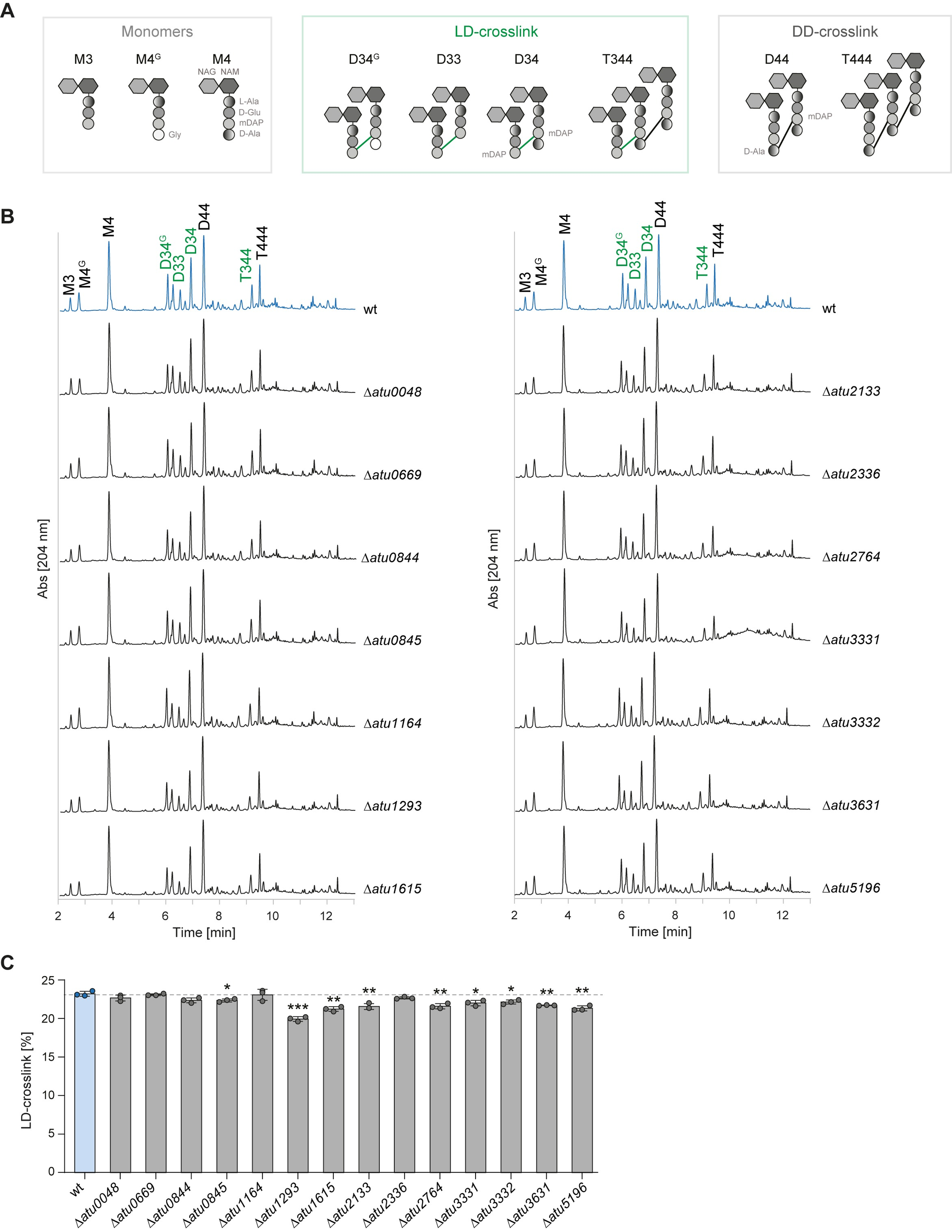

Supplement: S4 Fig — (A) Schematic structure and nomenclature of the muropeptides and crosslink type. Muropeptide structures are shown in S1 Table. (B) Representative UV muropeptide profiles of A. tumefaciens wt and single Δldt mutants grown in LB5. The major peaks are indicated. LD-crosslinked muropeptides are labeled in green. (C) Relative abundance of LD-crosslinked dimers in A. tumefaciens wt and single Δldt mutants grown in LB5. Error bars represent standard deviation. *, p <0.05; **, p <0.01; ***, p <0.001. (TIF) [file pgen.1011449.s004.tif]

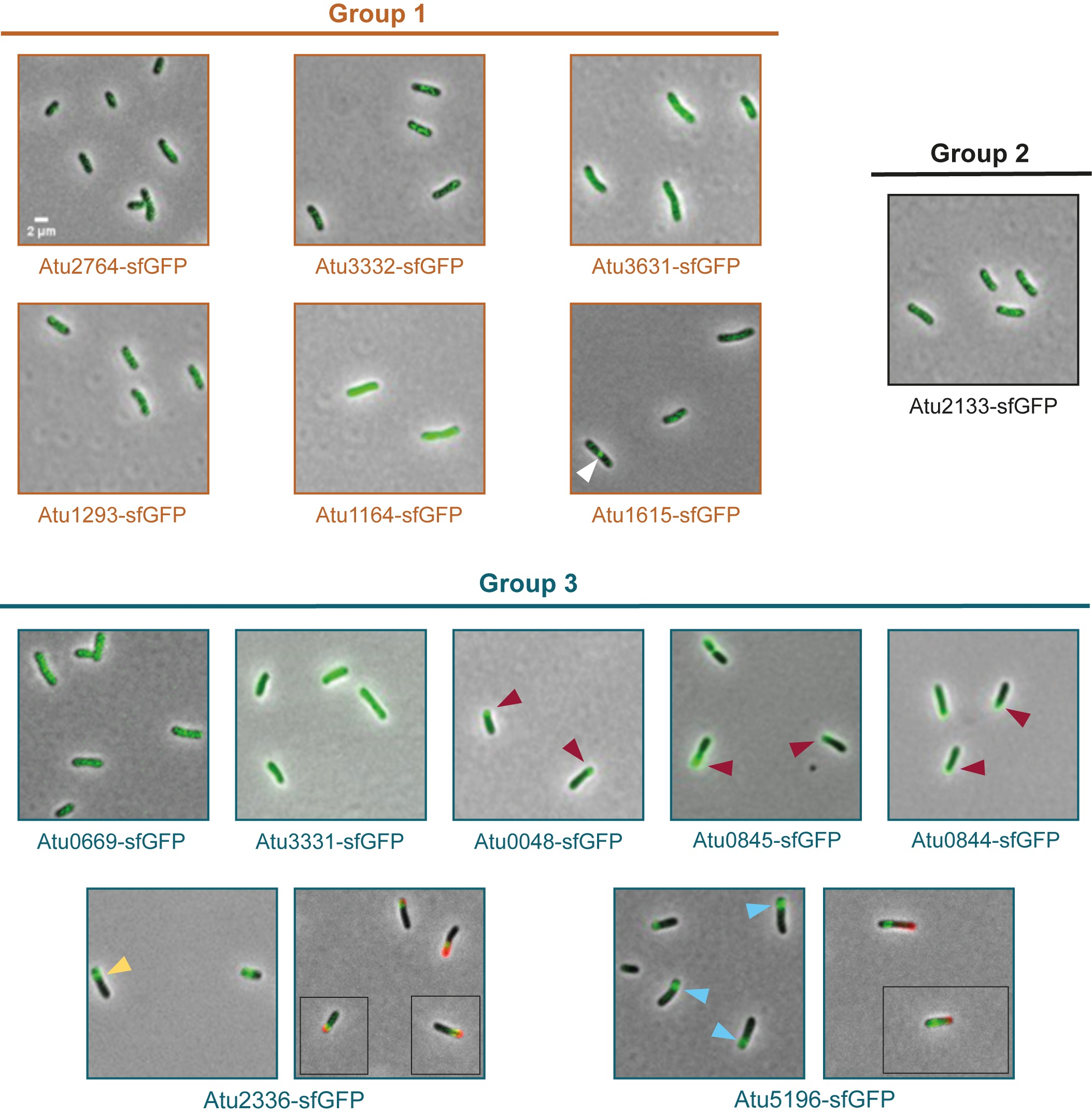

Supplement: S5 Fig — LDTs were fused to superfolder GFP (sfGFP) and expressed from a plasmid in wild type cells. Group 1 and 2 LDTs generally exhibit disperse localization, with Atu1615-sfGFP exhibiting mid-cell localization (white arrowhead). Members of group 3 have differential localization: Atu0048, Atu0844, and Atu0845 have strong growth pole localization (maroon arrowhead); Atu2336 localizes in a subpolar region adjacent to the growth pole (left panel, gold arrowhead) as shown when dual labeled with TADA (right panel, red fluorescence) which indicates the growth pole; and Atu5196 localizes in a subpolar region near the old pole (left panel, blue arrowhead) as shown when dual labeled with TADA (right panel, red fluorescence). (TIF) [file pgen.1011449.s005.tif]

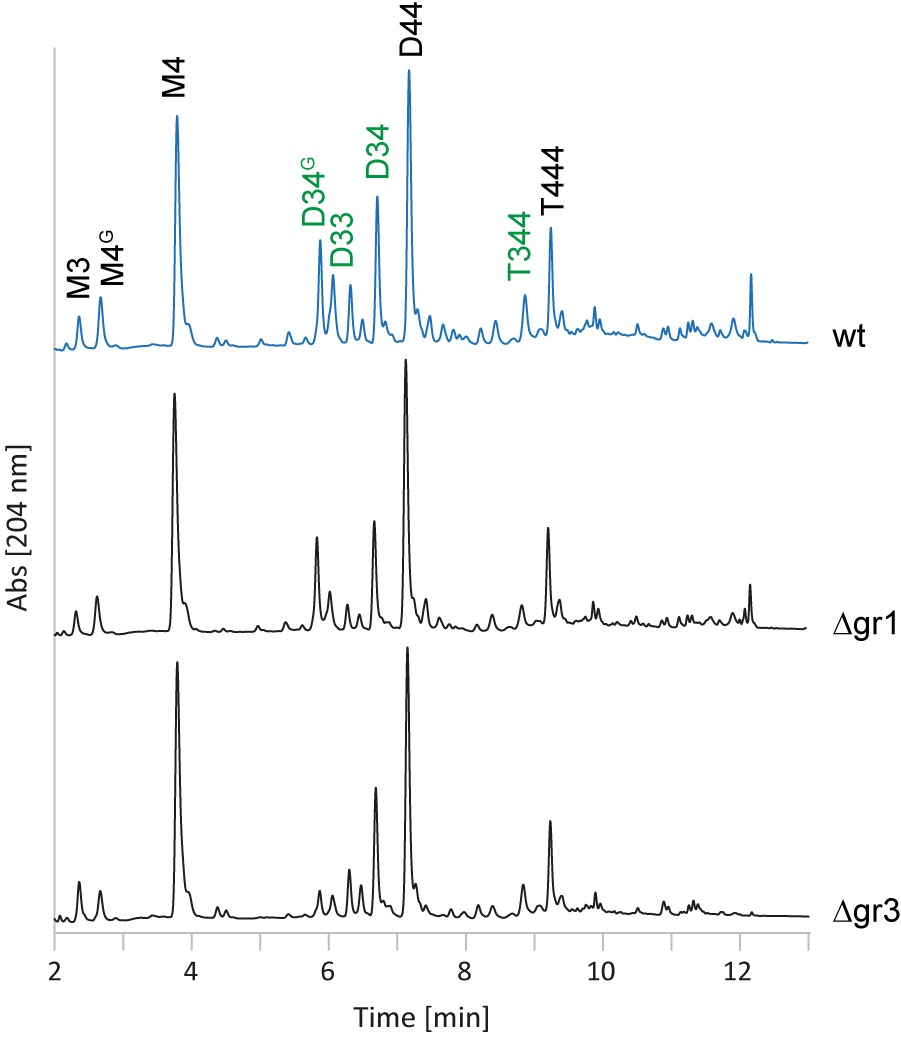

Supplement: S6 Fig — Representative UV muropeptide profiles of A. tumefaciens wild type (wt), Δgr1 and Δgr3 strains grown in LB5. The major peaks are indicated. LD-crosslinked muropeptides are labeled in green, muropeptide structures are shown in S1 Table. (TIF) [file pgen.1011449.s006.tif]

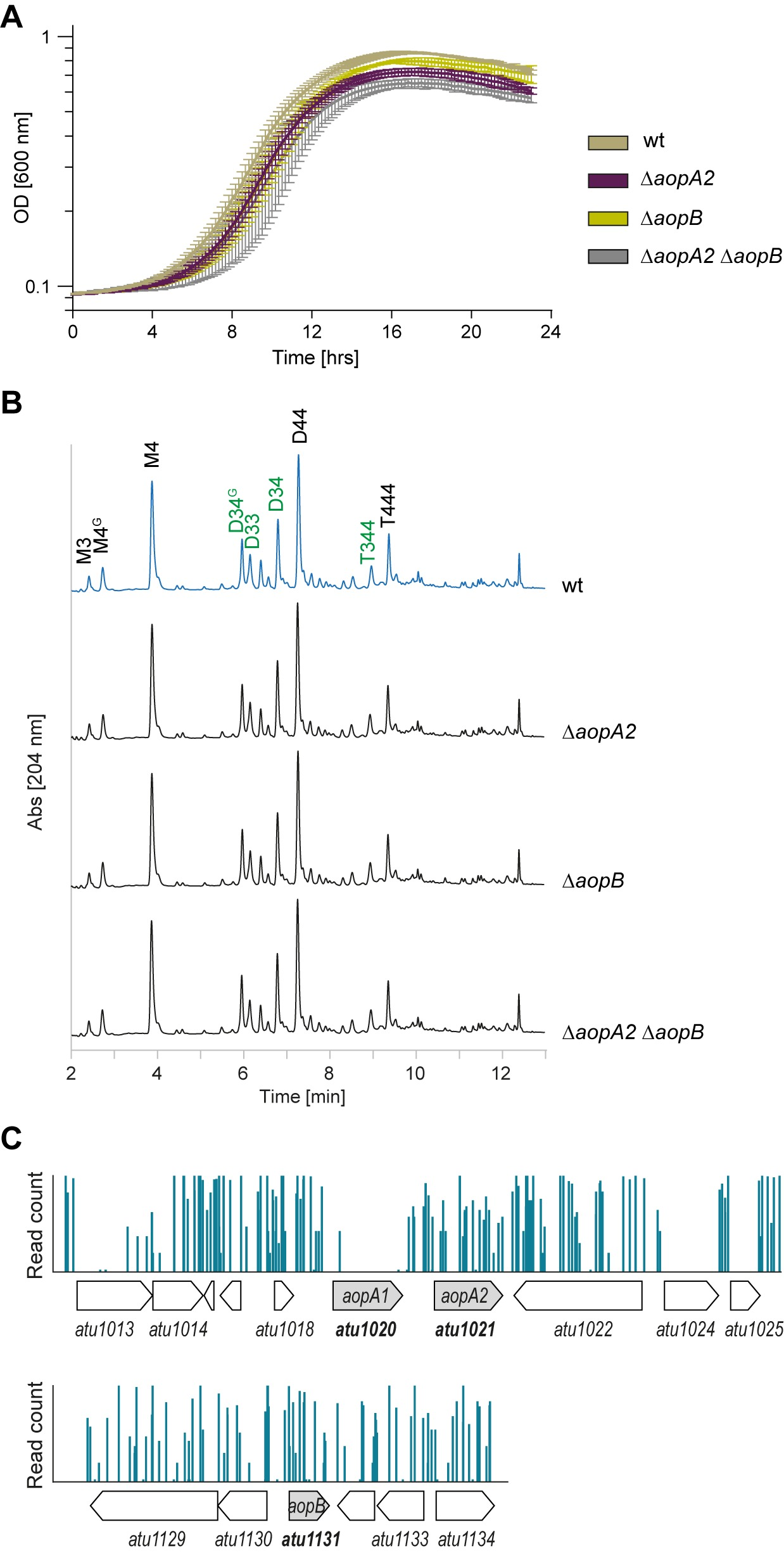

Supplement: S7 Fig — (A) Growth curves of A. tumefaciens wild type (wt) and OMP mutants in LB5 (5% NaCl) medium. (B) Representative UV muropeptide profiles of A. tumefaciens wild type (wt) and the indicated OMP mutant strains grown in LB5. The major peaks are indicated. LD-crosslinked muropeptides are labeled in green, muropeptide structures are shown in S1 Table. (C) Assessment of gene essentiality by Tn-seq. The plots show the read counts for transposon insertions in the OMP and neighbor genes in the WT strain. (TIF) [file pgen.1011449.s007.tif]

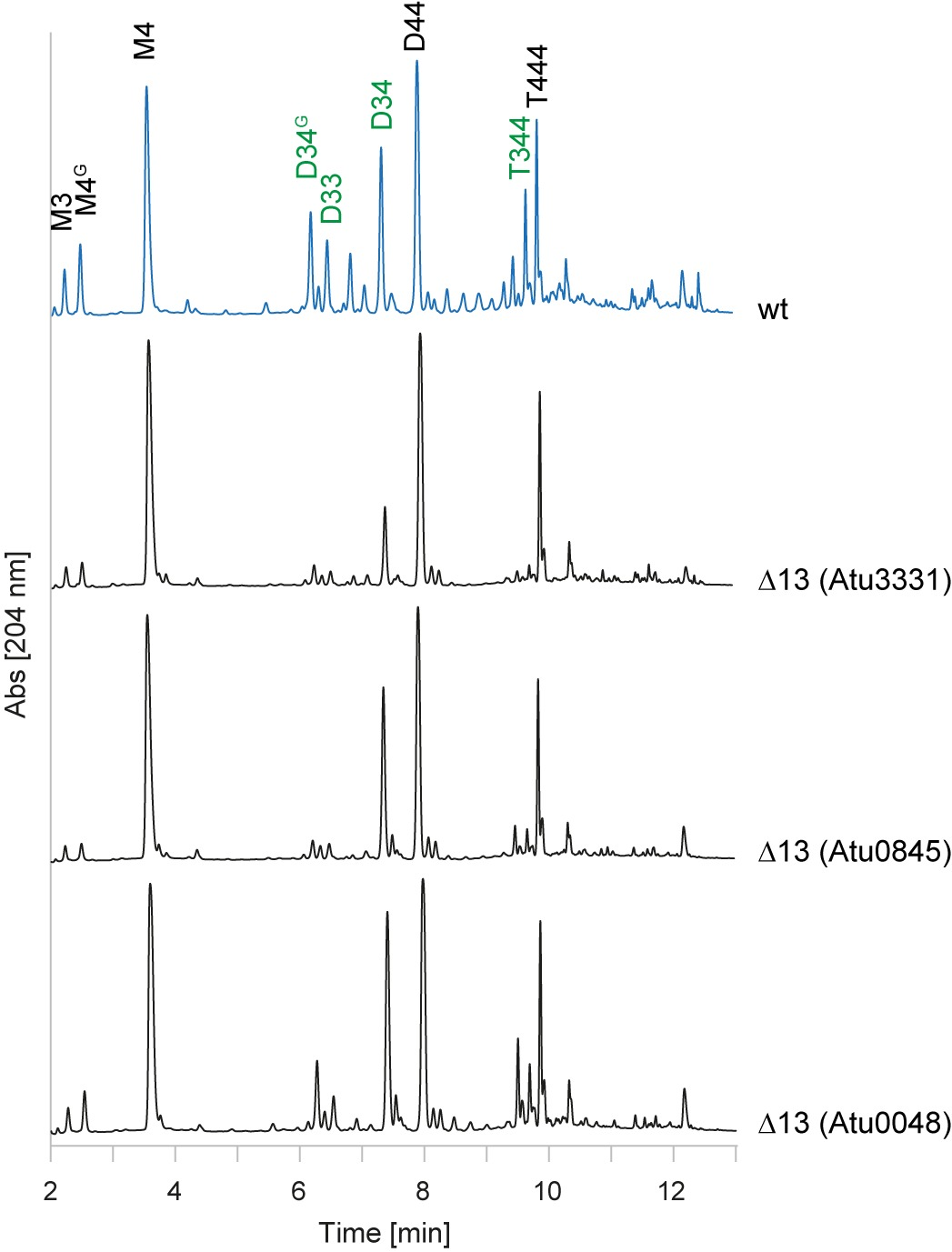

Supplement: S8 Fig — Representative UV muropeptide profiles of A. tumefaciens wild type (wt) and Δ13 ldts mutant strains grown in LB5. The major peaks are indicated. LD-crosslinked muropeptides are labeled in green, muropeptide structures are shown in S1 Table. (TIF) [file pgen.1011449.s008.tif]

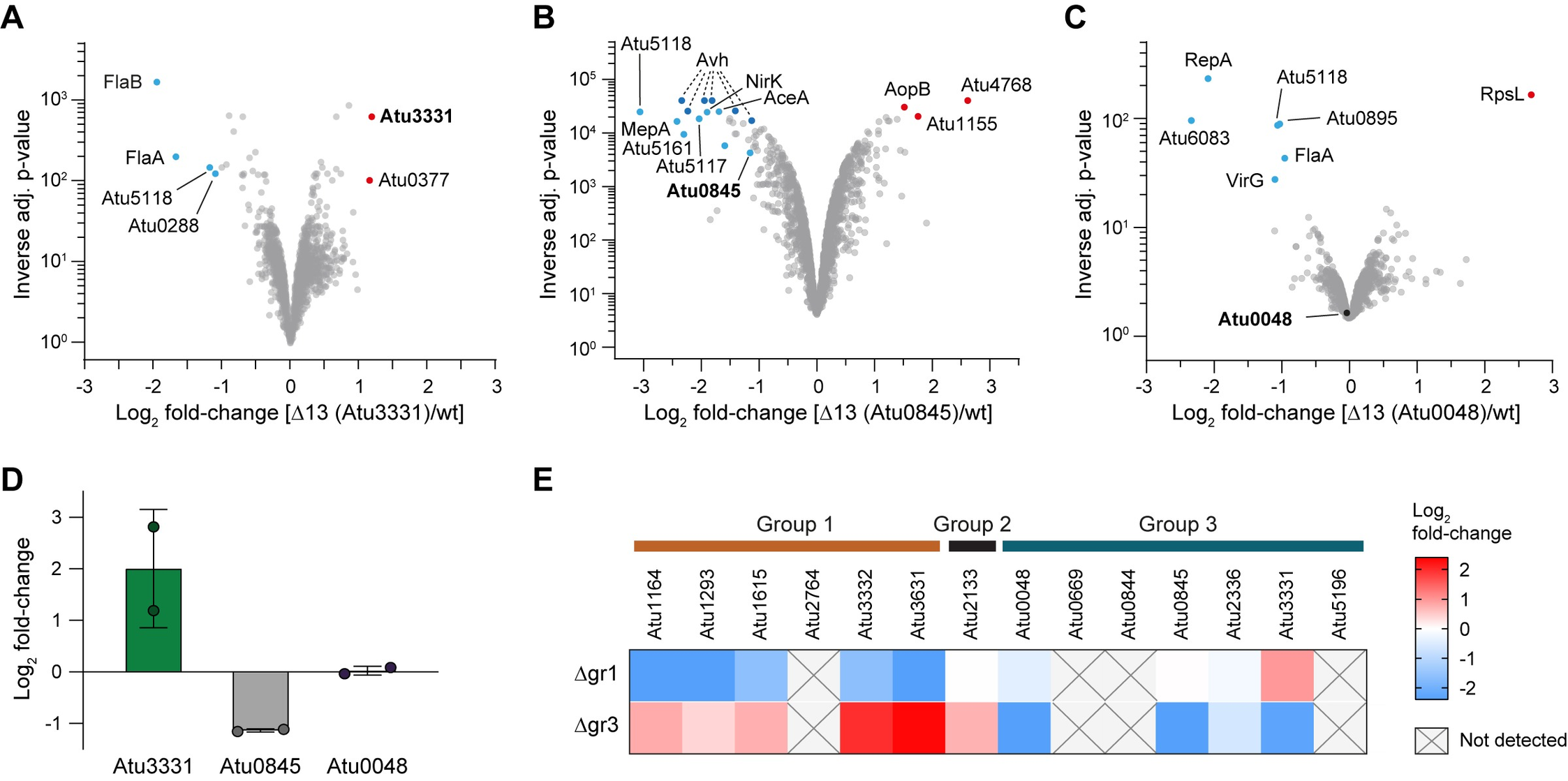

Supplement: S9 Fig — Volcano plot depicting the ratio of protein abundance of the A. tumefaciens Δ13 (Atu3331) (A), Δ13 (Atu0845) (B) and the Δ13 (Atu0845) (C) mutants relative to wild type. Proteins shown in blue and red have significantly lower and higher abundance, respectively. Data is presented in S4 Table. (D) Relative abundance of the remaining LDT in the corresponding Δ13 ldts mutant strains. (E) Relative abundance of all LDTs in the Δgr1 and Δgr3 mutant strains. (TIF) [file pgen.1011449.s009.tif]

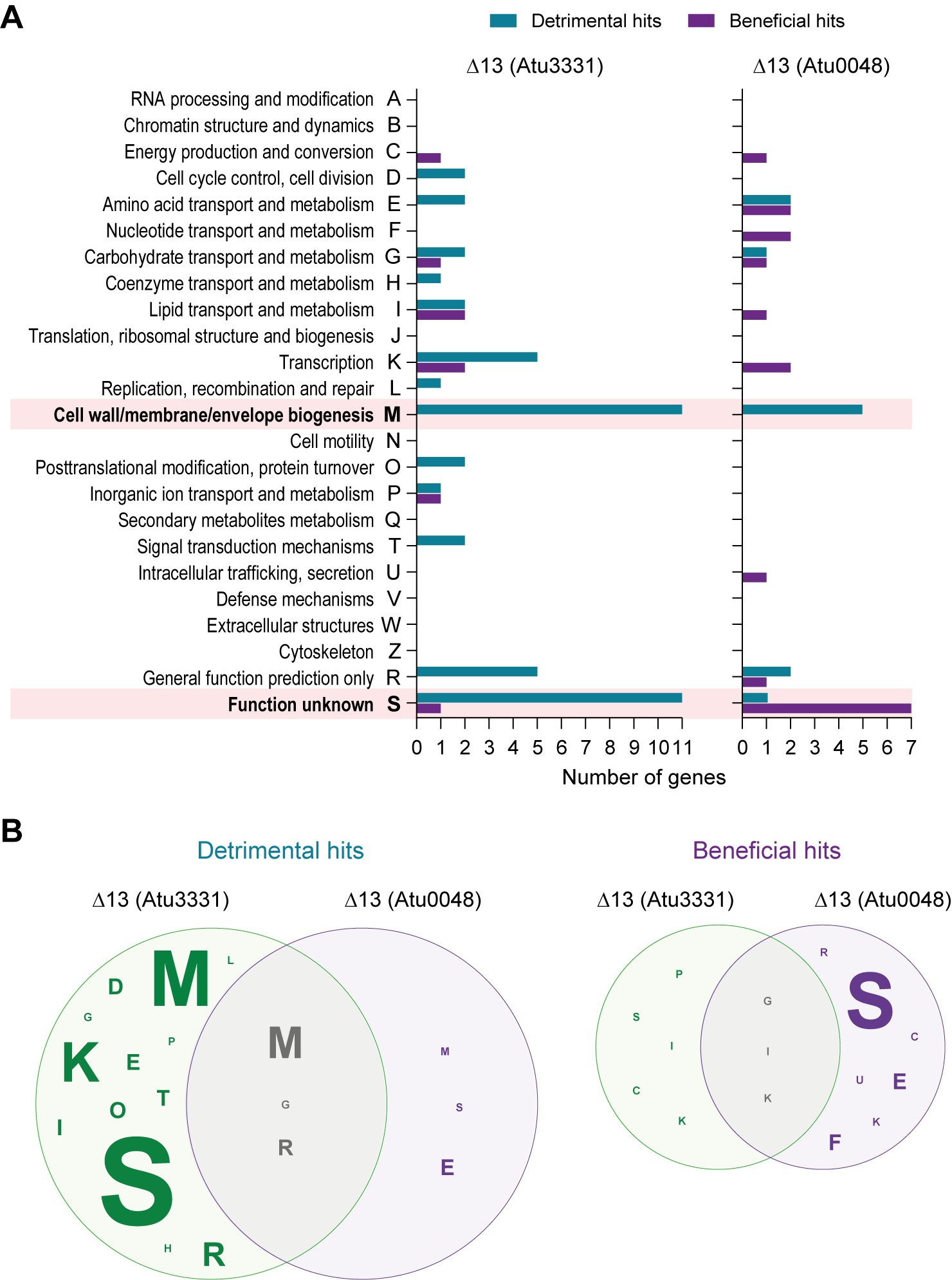

Supplement: S10 Fig — (A) Protein functions (COG functional classification) of the significantly synthetically detrimental and synthetically beneficial hits in the A. tumefaciens Δ13 (Atu3331) and Δ13 (Atu0048) mutant strains. (B) Venn diagrams representing the overlap of protein functions between the significantly synthetically detrimental (left) and synthetically beneficial (right) hits in the A. tumefaciens Δ13 (Atu3331) and Δ13 (Atu0048) mutant strains. The size of the letter is proportional to the number of genes within the specific COG functional classification. (TIF) [file pgen.1011449.s010.tif]

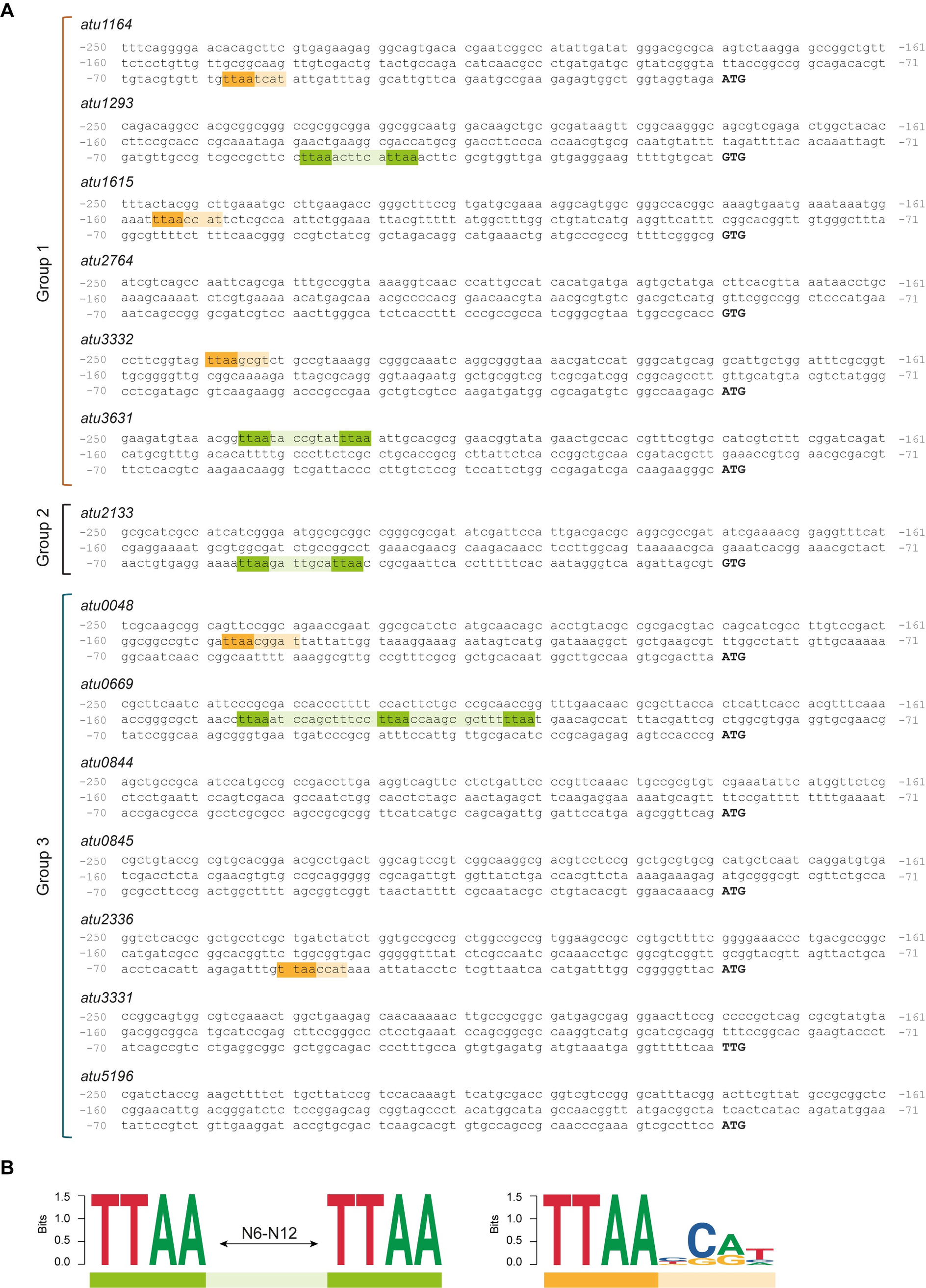

Supplement: S11 Fig — (A) Position of the consensus TTAA-N7-TTAA binding sites with atypical (N ≠ 7) spacing and atypical TTAACCAT motif are displayed in green and orange boxes, respectively. (B) Sequence logos of the atypical CtrA-binding motifs found. (TIF) [file pgen.1011449.s011.tif]
